# Network Dynamics in Elemental Assimilation and Metabolism

**DOI:** 10.3390/e23121633

**Published:** 2021-12-04

**Authors:** Austen Curtin, Christine Austin, Alessandro Giuliani, Manuel Ruiz Marín, Francheska Merced-Nieves, Martha M. Téllez-Rojo, Robert O. Wright, Manish Arora, Paul Curtin

**Affiliations:** 1 Department of Environmental Medicine and Public Health, Icahn School of Medicine at Mount Sinai, One Gustave L Levy Place, Box 1057, New York, NY 10029, USA; austen.curtin@mssm.edu (A.C.); christine.austin@mssm.edu (C.A.); francheska.merced-nieves@mssm.edu (F.M.-N.); robert.wright@mssm.edu (R.O.W.); manish.arora@mssm.edu (M.A.); 2 Environment and Health Department, Istituto Superiore di Sanità, 00161 Rome, Italy; alessandro.giuliani@iss.it; 3 Departamento de Métodos Cuantitativos, Universidad Politécnica de Cartagena, 30202 Cartagena, Spain; manuelruiz.spain@gmail.com; 4 Center for Nutrition and Health Research, National Institute of Public Health, Cuernavaca 62100, Morelos, Mexico; mmtellez@insp.mx

**Keywords:** recurrence quantification analysis, network analysis, graph theory, elemental metabolism, environmental exposures

## Abstract

Metabolism and physiology frequently follow non-linear rhythmic patterns which are reflected in concepts of homeostasis and circadian rhythms, yet few biomarkers are studied as dynamical systems. For instance, healthy human development depends on the assimilation and metabolism of essential elements, often accompanied by exposures to non-essential elements which may be toxic. In this study, we applied laser ablation-inductively coupled plasma-mass spectrometry (LA-ICP-MS) to reconstruct longitudinal exposure profiles of essential and non-essential elements throughout prenatal and early post-natal development. We applied cross-recurrence quantification analysis (CRQA) to characterize dynamics involved in elemental integration, and to construct a graph-theory based analysis of elemental metabolism. Our findings show how exposure to lead, a well-characterized toxicant, perturbs the metabolism of essential elements. In particular, our findings indicate that high levels of lead exposure dysregulate global aspects of metabolic network connectivity. For example, the magnitude of each element’s degree was increased in children exposed to high lead levels. Similarly, high lead exposure yielded discrete effects on specific essential elements, particularly zinc and magnesium, which showed reduced network metrics compared to other elements. In sum, this approach presents a new, systems-based perspective on the dynamics involved in elemental metabolism during critical periods of human development.

## 1. Introduction

There are myriad examples in biology of rhythmic patterns of physiology. Sleep occurs on a 24 h cycle. The hormone cortisol follows a diurnal pattern. Menstrual cycles are approximately monthly. Disruptions in these cyclic biological processes clearly affect health but are difficult to quantify using linear or even non-linear regression analysis. To capture the impact of environmental factors that disrupt homeostasis, different approaches are likely needed to capture patterns of rhythm that predict and reflect health. As an example, children are persistently exposed to chemical elements throughout their pre- and post-natal development, many of which are essential to the emergence of healthy development and metabolic function [1]. Elements considered essential to healthy development include zinc (Zn), copper (Cu), magnesium (Mg), and manganese (Mn), although the impact of these chemicals in supporting healthy development may be dependent not just on dose, but also on the timing of exposures. Trace elements are mediated through in utero development via the placenta, and are accordingly dependent on maternal diet, experience, and exposure history [2,3]. Post-natal levels are similarly mediated by parental influences, and, ultimately, by children’s diet and environment [4,5]. These factors, and concomitant exposures to non-essential and toxic elements, are typically studied by the assessment of exposure biomarkers in blood or urine, which can capture a momentary “snapshot” of a child’s level of exposure [6]. Recent innovations in exposure assessment, primarily focusing on the analysis of shed deciduous (“baby”) teeth, allow for the reconstruction of longitudinal biomarker profiles which capture the time-varying concentration of exposure biomarkers throughout prenatal and postnatal development at a time scale that allows for rhythmic patterns to be quantified [6,7].

Several recent studies have approached the analysis of longitudinal elemental biomarkers through the lens of recurrence quantification analysis (RQA) and a related bivariate method, cross-recurrence quantification analysis (CRQA). Key insights developed from this approach include the identification of periodic dynamics, indicative of seasonal, circaseptan, or circadian metabolic cycles, in the metabolism of essential elements [8], and the disruption of these processes in the emergence of neurodevelopmental disorders [9,10]. Critically, although these insights have been essential in highlighting the importance of metabolic dynamics in health and disease, the focus of these studies on single- and dual-element dynamics has not extended to the consideration of broader network dynamics; that is, although these approaches effectively characterize dynamics in discrete elemental pathways, or in pairwise combinations of pathways, they have not considered the overall architecture of dynamic connectivity.

Similarly, several studies have looked at environmental exposures with network analyses, though these studies have typically focused on levels of organization beyond exposure metabolism. Social network architecture, for example, is a common focus of analysis in environmental epidemiological studies [11,12], either as a mediator of exposure or relating to the consequences of exposure [13,14]. Similarly, network analysis is a powerful tool for exploring the organization of molecular mechanisms, particularly genomic and epigenomic mechanisms [15,16], and the modulation of these factors via exposure. Network analysis of environmental exposure and metabolism remain relatively unexplored in the current epidemiological literature, although with some notable exceptions, such as the recent work by Li et al. [17], which utilized a hierarchical community network to integrate exposomic and metabolomic factors.

Here, we developed an approach to characterize elemental metabolism through the lens of network connectivity; that is, the temporal correlation structure among different elements. Using tooth-based biomarkers which provide longitudinal measurements of elemental exposure, we applied CRQA to characterize dynamic connectivity within and between essential and non-essential elements, including zinc, copper, manganese, magnesium, strontium, barium, and lithium. Similarly, we characterized exposure to an unambiguous toxicant, lead. We highlight the utility of this approach in a use case developed to address three basic and/or applied research questions. First, in considering the architecture of dynamic connectivity, we contrast the role of discrete elemental pathways in the overall metabolic network. Second, in consideration of lead exposure, we tested how lead impacts global network architecture. Finally, we extend this analysis to consider the effects of lead on discrete elemental pathways. In sum, the approach developed here presents a new approach to characterize elemental exposure and metabolism in terms of network architecture and connectivity, providing biomarkers directly impinging on the changes in global metabolism structure rather than single chemical species.

## 2. Materials and Methods

### 2.1. Study Population

Human study participants were recruited from the Programming Research in Obesity, Growth, Environment, and Social Stressors (PROGRESS) cohort in Mexico City, Mexico. Pregnant women who were receiving health insurance and prenatal care through the Mexican Social Security Institute (Instituto Mexicano del Seguro Social [IMSS], Ciudad de México, Mexico) were recruited between July 2007 and February 2011. The IMSS provides health care to affiliated private sector employees, most of whom are low- to middle-income workers, and their families. In total, 434 participating children provided deciduous tooth samples for this analysis.

### 2.2. Laser Ablation-Inductively Coupled Plasma-Mass Spectrometry

Our approach to measuring metals in teeth using laser ablation-inductively coupled plasma-mass spectrometry (LA-ICP-MS) and assigning developmental times has been detailed elsewhere [7,18]. Briefly, teeth are sectioned and the neonatal line (a histological feature formed in enamel and dentine at the time of birth) and incremental markings are used to assign temporal information to sampling points. A New Wave Research NWR-193 (ESI, Fremont, CA, USA) laser ablation unit equipped with a 193 nm ArF excimer laser was connected to an Agilent Technologies 8800 triple-quad ICP-MS (Agilent Technologies, USA). Helium was used as a carrier gas from the laser ablation cell and mixed with argon via a Y-piece before introduction to the ICP-MS. The system was tuned daily using NIST SRM 612 (trace elements in glass) to monitor sensitivity (maximum analyte ion counts), oxide formation (^232^Th^16^O^+^/^232^Th^+^, <0.3%) and fractionation (^232^Th^+^/^238^U^+^, 100 ± 5%). The laser was scanned in dentine parallel to the enamel-dentine junction from the dentine horn tip towards the tooth cervix. A pre-ablation scan was run to remove any surface contamination. Data were analyzed as metal to calcium ratios (e.g., ^138^Ba:^43^Ca) to control for any variations in the mineral content within a tooth and between samples. Exposure profiles covered a range from −143 days prenatally to 389 days postnatally.

### 2.3. Recurrence Quantification Analysis

We previously described the application of recurrence quantification analysis (RQA) and cross-recurrence quantification analysis (CRQA) to characterize dynamics in longitudinal tooth biomarkers in prior studies [8,9,10,19]. Briefly, this non-linear analytical method involves the application of Taken’s delay embedding for attractor reconstruction; a threshold function, ε, is then applied to each point in the reconstructed attractor, and the timing of the system’s reentry within this perimeter is defined as a recurrence. RQA focuses on the autocorrelation structure of a time series, whereas CRQA describes the relation between two different time series. Accordingly, three parameters require specification in the reconstruction of the attractor: ε, which as noted indicates the size of the specified threshold; *m*, which indicates the dimensionality of the reconstructed system; and τ, which indicates the delay (lag) used to define lag-delayed dimensions. To determine an appropriate delay, a mutual information algorithm was used to estimate the effect of varying lags on mutual information between the original and delayed signals; the minimal lag interval which minimized mutual information was used as τ. Similarly, to estimate *m*, a false nearest neighbors algorithm was used, and the dimensionality chosen was likewise the *m*-value which minimized the number of false nearest neighbors. For specifying ε, two approaches were taken. First, in our initial analysis, which focused on the analysis of recurrence rates (in some senses, a non-linear proxy of correlation), ε was approximated by taking 10% of the estimated phase space diameter of reconstructed attractors. In subsequent analyses, which focused on other RQA/CRQA metrics, the value of ε was varied in our analysis to yield a fixed recurrence rate of 0.1 in order to facilitate cross-element comparisons (that is, in cases where recurrence rates might vary, but our interest was in features such as entropy). A fixed recurrence rate of 0.1 was chosen to align our results with prior investigations, and because this value approximated the mean recurrence rate across all elements (0.103) when estimated using a ε-function based on phase space diameter. Following the specification of these parameters, RQA and CRQA were used to calculate standard metrics of signal dynamics, particularly entropy in diagonal lines, which, in the context of a recurrence plot, indicate the emergence of periodicity within and between signals. C/RQA analyses were performed with the Cross-Recurrence Toolbox v5.16 (http://tocsy.pik-potsdam.de/CRPtoolbox/ (accessed on 19 March 2021)) in Matlab v2019b (Mathworks) and the Dynamical Systems package in Julia (https://juliadynamics.github.io/DynamicalSystems.jl/latest/ (accessed on 19 March 2021)).

### 2.4. Graph Construction and Network Analysis

Following the application of CRQA to characterize dynamics between longitudinal elemental time series, these features were used to construct and quantify network dynamics. In our initial analysis, focusing on the quantification of recurrence rates (RR), cross-recurrence rates between elements were used as edges in the construction of graphs. Recurrence rates which were below the median recurrence rate were excluded from this analysis; graphs thus reflect only non-trivial connections between elements. This approach was separately applied to each individual in the study, yielding, per subject, a unique graph of cross-element dependencies. We then applied standard graph theory analytical methods to quantify key metrics of connectedness among each individuals’ metabolic graph, including the degree [20], betweenness [21], closeness [22], eigenvalue, (local) clustering coefficient [23], and eccentricity [24] of connections for each element. Although our initial analysis used recurrence rates to define edges between elements, our subsequent analysis utilized entropy in diagonal lengths to define edges; the analytical procedure in both approaches was identical.

### 2.5. Statistical Analysis

The application of CRQA to quantify cross-element metabolic dynamics, and the usage of graph theory to characterize, in each individual, the role of discrete elements, provided an array of descriptive statistics for each individual. To test if toxic exposures, in particular lead exposure, impacted these metrics, we constructed linear models to test for associations between high/low lead exposure related to network dynamics for each essential (Cu, Mg, Mn, Zn) and non-essential (Ba, Li, Sr) element. For each of the 6 graph theory metrics derived, we constructed a discrete linear model to determine if that feature varied by element, by Pb exposure, or by the interaction of these functions, such that:

Centrality = α + βElement + βPb + βElement × Pb,(1)

where “Centrality” is an example of one derived network measure, α is the model intercept, β represents regression parameters, “Element” indicates a categorical (dummy-coded) factor representing a given element, and Pb represents a dichotomized factor for lead exposure. To account for repeated measurements in participants—that is, multiple elements were measured in each participant—a random effect was included in the construction of linear mixed models. In sum, these models yield direct hypothesis tests to answer the questions: Are some elements more connected than others? Does lead exposure impact connectivity? And, are lead’s effects specific to some elements?

## 3. Results

### 3.1. Network Dynamics in Recurrence Rates

**Figure 1:**
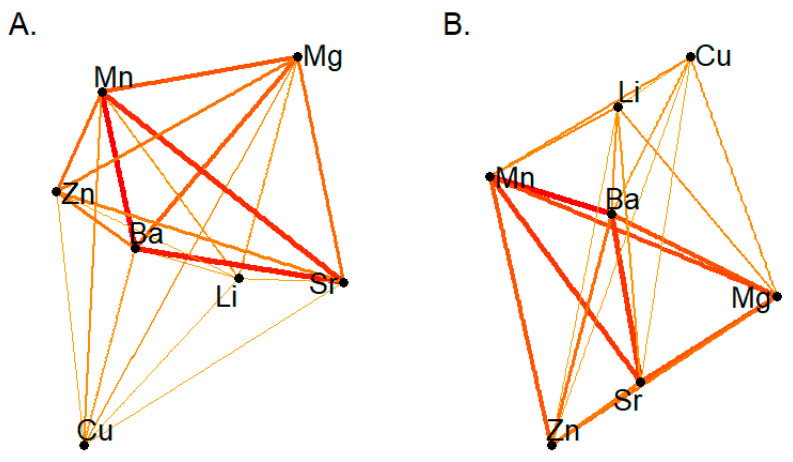
Network architecture in children exposed to low (**A**) or high (**B**) levels of lead. Edges reflect the mean recurrence rates observed between elements in each group. In subsequent analyses, unique networks were constructed in each participant, excluding edges with recurrence rates below the median global recurrence rate, in order to contrast connectivity between elements and between high- and low-lead conditions.

Our initial analysis focused on the characterization of networks defined by recurrence rates in cross-recurrence quantification analysis (CRQA), a metric which essentially captures non-linear dependencies (correlations) between elements. For each participant, a unique network was constructed such that each discrete element in this analysis served as a node in the network, and edges were defined by the CRQA recurrence rates; from each network, six graph metrics were derived to characterize connectivity between elements in these networks. Figure 1 provides a high-level summary of this approach, with networks constructed for participants in low- and high-lead exposure conditions. In these depictions, edges were constructed by taking the mean (for that group) CRQA recurrence rates; however, in all analyses to follow, unique networks were constructed for each participant.

**Figure 2:**
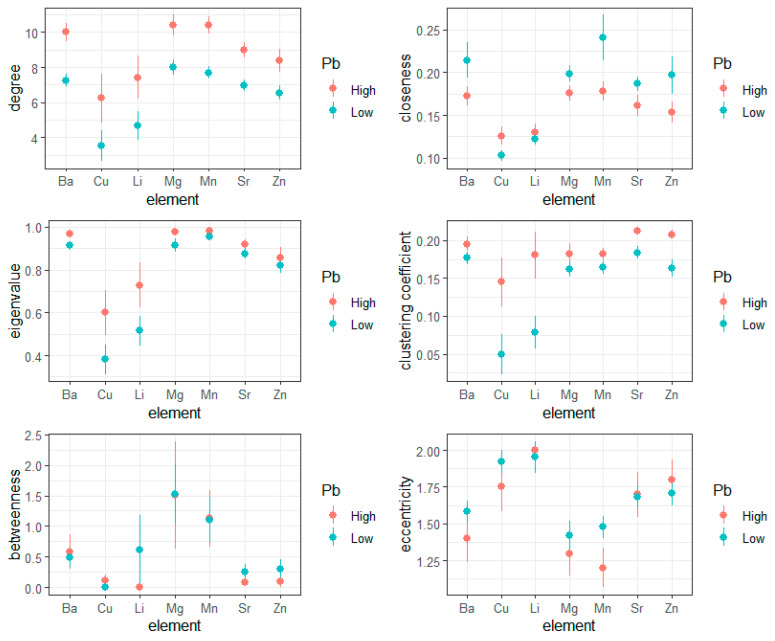
Recurrence rates in metabolic networks in children with high or low lead exposure. For each element (x-axis), graph theory metrics (y-axis) were estimated, with graph edges defined by recurrence rates estimated with cross-recurrence quantification analysis.

In Figure 2, we show graph theory metrics derived through the analysis of networks in children with high or low lead exposure. In Table 1 and Table 2, we provide details on hypothesis tests relating to the main effects of lead across all elements, or the discrete effects of lead within a given elemental pathway, respectively.

Generally, these results emphasize three general findings. First, irrespective of lead exposure, we find that the network connectivity varies significantly between different elements; copper and lithium, for example, consistently differed from other elements in their network connectivity. Second, relating to lead exposure, we find that network architecture is highly sensitive to toxicant exposure. The magnitude of each element’s degree was significantly elevated in children with high lead exposure, indicating that the lead exposure disrupts pathway-specific connectivity profiles. With other measures, for example closeness centrality, this trend was reversed, such that high lead exposure was associated with reduced closeness, i.e., lead yields reduced efficiency of information transmission between elements; however, this overall trend (main effect of lead) was not significant. Third, and most commonly, we find that the effects of lead exposure yield both network-wide and element-specific effects; that is, the magnitude of lead’s effects on network connectivity varies between elements. This effect was particularly prominent in measures of eigenvalue centrality and per-element clustering coefficients, where we observed that copper and lithium were significantly dysregulated by lead exposure, but other elements were not; we likewise found that zinc clustering coefficients were also significantly elevated with high lead exposure (*p* = 0.04).

### 3.2. Entropy in Elemental Metabolic Networks

**Figure 3:**
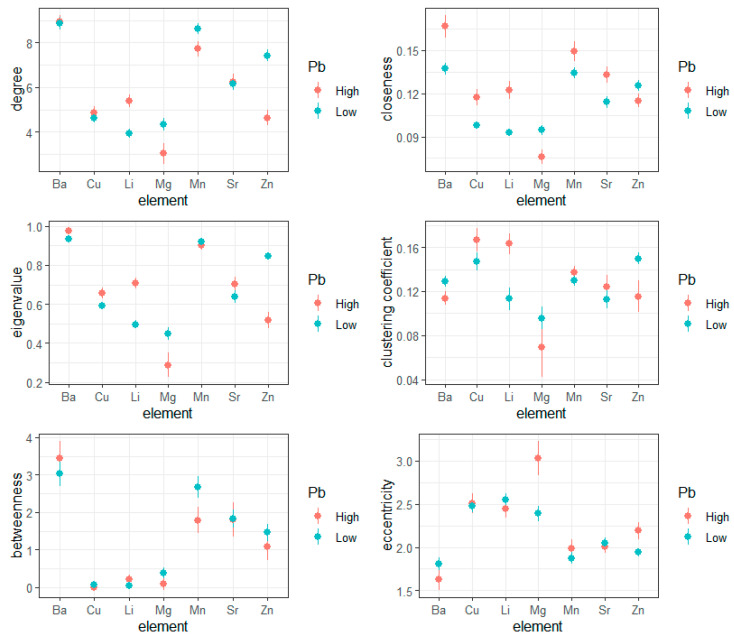
Entropy in metabolic networks in children with high or low lead exposure. For each element (x-axis), graph theory metrics (y-axis) were estimated, with graph edges defined by periodic entropy (that is, with respect to diagonal lines) estimated with cross-recurrence quantification analysis.

To understand the dynamics driving the observed differences in global connectivity metrics, we next undertook an analysis of periodic entropy derived from cross-recurrence quantification analysis (CRQA). As in our analysis of global correlation structure, for each individual we constructed a discrete metabolic network, here utilizing entropy emerging in between-element periodicity in the construction of edges. Results of these analyses, summarized across individuals, are shown for children with high and low lead exposure in Figure 3. In Table 3 and Table 4, we provide details on hypothesis tests relating to the main effects of lead across all elements, or the discrete effects of lead within a given elemental pathway, respectively.

Consistent with our analysis of recurrence rates, our findings in the analysis of entropy-based network connectivity indicate both general and discrete effects of lead exposure on metabolic network connectivity. For measures of general connectivity, e.g., number of edges, we found that high lead exposure did not yield a broadly consistent effect; rather, we found that high lead exposure was associated with discrete effects for different elemental pathways. In particular, lead exposure yielded significantly reduced connectivity in critical essential elements including zinc, magnesium, and manganese; conversely, high lead exposure increased the degree of connectivity associated with lithium.

For other measures of network architecture, particularly network closeness, we found high lead exposure elevated network closeness, indicating inefficiency in transmission among elements. However, even in the context of this broad pattern, we observed exceptions, such that zinc and manganese closeness were significantly reduced with high lead exposure.

Measures of networks centrality, including betweenness, eigenvalue centrality, and clustering coefficient, similarly experienced complex effects relating to lead exposure. For betweenness and eigenvalue centrality, these effects were manifest as borderline-significant main effects of high lead exposure (*p*-values approximately 0.05; see Table 3); or, in the case of clustering coefficients, significant main effects (*p* = 0.03), such that high lead exposure was generally associated with increased clustering coefficients. As in other cases, however, these effects also varied in direction and intensity across different elemental pathways. Betweenness centrality was significantly reduced in manganese and tended to differ between groups (*p*-values approximately 0.05; see Table 4) for zinc and barium. For eigenvalue centrality, high lead exposure was associated with reduced centrality in zinc and magnesium pathways, but was associated with increased centrality in all other elements. This general pattern was also consistent in comparing clustering coefficients.

Measures relating to path integration, including closeness and eccentricity, similarly reiterated the general pattern of global and discrete effects associated with lead exposure. For closeness, we observed that high lead exposure yielded increased closeness for barium, copper, lithium, manganese, and strontium, but, conversely, reduced closeness for the essential elements of magnesium and zinc. Similarly, with network eccentricity, we found that high lead exposure reduced eccentricity in barium connectivity, but conversely increased eccentricity for magnesium and zinc.

## 4. Discussion

Here we integrated cross-recurrence quantification analysis (CRQA) in a graph-theory based network analysis to characterize the effects of toxic elemental exposures on the assimilation and metabolism of essential elements. We used CRQA to construct network edges which captured global non-linear correlation structure, via recurrence rates, and, via the analysis of cross-element entropy, the complexity of periodic dynamics between varying elemental pathways. Our results identify three general principles. First, we show that discrete elemental pathways exhibit distinct network architectures, such that each element was associated with varying levels of degree, centrality, and path complexity. Second, we show that these features are highly sensitive to lead exposure, in that these features differ between children exposed to high and low levels of lead. Third, we show that the effects of lead exposure yield varying effects on different elemental pathways. In particular, we show that lead exposure dysregulates network architecture involving the essential elements of zinc, copper, magnesium, and manganese, and, similarly, also acts on non-essential elements including barium, strontium, and lithium. In sum, these results emphasize the utility of integrating dynamical analytical methods in network-based analyses to characterize the architecture of metabolic dynamics, and the perturbation of these processes following toxicological insult.

This paper follows several recent advances [8,9,10,20] which highlighted the utility of recurrence quantification analysis (RQA) and CRQA in characterizing dynamics involved in elemental exposure and metabolism. In contrast to those papers, which focused on the analysis of single- or dual-element dynamics, in this paper we characterize elemental dynamics through the lens of network connectivity. From this perspective, the critical descriptive measures analyzed here are the role of a given element in relation to the full array of elements measured. This provides an important conceptual and methodological advance in the consideration of elemental metabolism. Our findings nonetheless align well with those of prior studies. For example, prior studies have found that zinc and copper dynamics are dysregulated in subjects diagnosed with autism spectrum disorder and attention deficit hyperactivity disorder [9,10]. Similarly, prior studies have also identified elevated lead levels in these subjects [25]. Our current findings, which emphasize that lead dysregulates the network architecture involving zinc metabolism, provide a bridge between these results, suggesting that lead exposure plays a role in the dysregulation of mechanisms associated with these diseases.

This approach to exploring high-dimensional environmental exposure data parallels and complements current approaches in the literature, particularly so-called ‘mixtures’ analyses [1,26,27]. These approaches typically employ forms of supervised dimensionality-reduction, particularly weighted quantile sum regression [28,29,30] and Bayesian kernel machine regression [31,32], to link the additive or multiplicative integration of multiple exposure biomarkers to a single health outcome. From this perspective, the complexity of correlation patterns observed among exposure variables is something of a nuisance, which, if uncontrolled, may impede estimation of associative effects. In contrast, the utilization of a network-based analysis, as applied here, leverages the structural organization of correlations among exposures as the fundamental unit of analysis. As such, this approach may provide a fruitful and complementary avenue of investigation in future environmental epidemiological studies, which may be adapted to cross-sectional studies through the construction of simple correlation-based edges, or be applied to longitudinal exposures data, as was demonstrated in the present study.

Furthermore, at a more basic level, our findings emphasize the utility of both network-based and dynamical analyses as tools for understanding longitudinal metabolic processes. We previously reported that essential elements, including those studied in the current study, exhibit characteristic periodic dynamics which are markedly different from the dynamics of toxicant exposures [8]. Here, we demonstrate that these dynamics contribute to a global network architecture which is perturbed through toxicological insult. The approach outlined in the present paper opens new avenues for future investigations, shifting the unit of analysis and interpretation of a given elemental pathway’s function from intrinsic dynamics to integrative functionality. In this pursuit, the flexibility of the paradigm developed here will be a key advantage for future studies, as assessment of net network dynamics can be linked to cross-sectional assessments of health, parameterized much as lead was dichotomized in the present study. Alternatively, given the availability of health indicators assessed in longitudinal contexts, such as electrophysiological or circadian assessment, RQA-based assessment of health outcome measures could be used to connect periodic dynamics across multiple stages of biological organization, i.e., from metabolic indicators to behavioral/organismal indicators.

## 5. Conclusions

In this study we introduced the use of cross-recurrence quantification analysis for applications in network analysis in elemental assimilation and metabolism. We demonstrate that these measures are highly sensitive indicators of toxicant exposures; here, in the case of high or low lead exposure. The approach advanced in this paper offers a new research tool and perspective for the exploration of exposure assimilation and metabolism, and future studies may expand on these findings through the application of this approach in contexts relating to health and disease.

From a more methodological perspective, these findings are still far from linking the observed changes in dynamic metabolic correlation structure to a mechanistic hypothesis. Instead, this approach should be viewed as a tool or paradigm that future studies can use to address those questions. For example, future studies might contrast differences in lead-related effects on network connectivity across genotypes relating to elemental metabolism. Similar approaches may be also useful in extending current theoretical perspectives and providing practical frameworks for hypothesis testing. For example, the current dominant paradigm in exposure-related research focuses on the Developmental Origins of Health and Disease (DoHAD) hypothesis, which focuses on the exploration of early-life conditions as antecedents of later-life health and disease [33]. A network-based perspective on early-life metabolism provides a framework for investigating how environmental programming of metabolic networks—as, here, we characterized the effects of lead exposure on the integrated metabolic function of essential elements—relates to the emergence of disease in later life. In practice, this can be implemented through a simple modification of the study design applied here; that is, rather than contrasting the effects of high and low lead exposure, future studies could compare metabolic networks in healthy participants and those who later experience disease. From the present findings, we can state that lead exposure, by increasing the degree, i.e., the ‘aspecific connectivity’ among elements, provokes a general stress on global metabolism, using the generalized increase in correlation as a signature of a stress response (e.g., see Gorban et al., 2021 [34]). As such, we can surely state that network dynamical biomarkers provide new means to study environment–physiology interaction.

## Figures and Tables

**Table 1:** Diffuse effects of lead exposure in networks constructed with recurrence rates ^1^.

Measure	*p*-Value
Degree	0.002
Closeness	0.301
Betweenness	0.895
Eigenvalue	0.372
Clustering Coefficient	0.375
Eccentricity	0.330

^1^ *p*-values reflect tests for the main effect of lead in linear mixed models which include covariates of element, lead, and element × lead interactions.

**Table 2:** Discrete effects of lead exposure in networks constructed with recurrence rates ^1^.

Element	Degree	Closeness	Betweenness	Eigenvalue	Clustering Coefficient	Eccentricity
Ba	0.002	0.301	0.895	0.372	0.375	0.330
Cu	0.001	0.325	0.882	0.007	<0.001	0.582
Li	0.002	0.334	0.432	0.006	<0.001	0.424
Mg	0.003	0.273	0.987	0.314	0.234	0.532
Mn	0.003	0.279	0.974	0.632	0.438	0.120
Sr	0.015	0.271	0.782	0.510	0.153	0.872
Zn	0.027	0.261	0.741	0.539	0.041	0.555

^1^ *p*-values reflect post-hoc tests for the comparison of high-lead vs. low-lead conditions within each discrete elemental pathway.

**Table 3:** Diffuse effects of lead exposure in networks constructed with periodic entropy ^1^.

Measure	*p*-Value
Degree	0.784
Closeness	0.000
Betweenness	0.052
Eigenvalue	0.057
Clustering Coefficient	0.034
Eccentricity	0.007

^1^ *p*-values reflect tests for the main effect of lead in linear mixed models which include covariates of element, lead, and element × lead interactions.

**Table 4:** Discrete effects of lead exposure in networks constructed with periodic entropy ^1^.

Element	Degree	Closeness	Betweenness	Eigenvalue	Clustering Coefficient	Eccentricity
Ba	0.784	<0.001	0.052	0.057	0.034	0.007
Cu	0.182	<0.001	0.731	0.002	0.006	0.640
Li	<0.001	<0.001	0.434	<0.001	<0.001	0.137
Mg	<0.001	0.341	0.399	<0.001	0.044	0.001
Mn	<0.001	<0.001	<0.001	0.341	0.321	0.107
Sr	0.734	<0.001	0.906	0.002	0.118	0.503
Zn	<0.001	0.022	0.066	<0.001	<0.001	<0.001

^1^ *p*-values reflect post-hoc tests for the comparison of high-lead vs. low-lead conditions within each discrete elemental pathway.

## Data Availability

Data sets generated and analyzed in the current study are not publicly available because they may contain potentially sensitive personal health information but may be available upon request to authors, pending approval of related Institutional Review Boards.
